# Characterization of Mitochondrial Genomes and Evolutionary Relationships in Two *Moenkhausia* (Characiformes: Acestrorhamphidae)

**DOI:** 10.1002/ece3.72968

**Published:** 2026-01-19

**Authors:** Cheng‐He Sun, Xiao‐Die Chen, Yi‐Jing Zhan, Yang Xu, Chang‐Hu Lu

**Affiliations:** ^1^ College of Life Sciences Nanjing Forestry University Nanjing China

**Keywords:** next‐generation sequencing, phylogenetic evolution, sequence analysis

## Abstract

The family Acestrorhamphidae, comprising fish species with high nutritional and ornamental value, continues to face significant controversies regarding their systematic classification and evolutionary relationships. Due to notable convergent evolution and intraspecific variation in morphological characteristics, traditional morphological methods struggle to achieve reliable taxonomic delineation. In this study, high‐throughput sequencing technology was employed to sequence and assemble the mitochondrial genomes of two species, *Moenkhausia rubra* and *Moenkhausia nigromarginata* , with lengths of 16,439 and 16,461 bp, respectively. Both genomes exhibit the typical mitochondrial gene structure of vertebrates, along with a distinct AT base bias and an anti‐G bias. A phylogenetic tree constructed based on the mitochondrial genomes of 41 closely related species revealed that the genus *Moenkhausia* is monophyletic, with *M. nigromarginata* and *M. rubra* clustering together, indicating a close genetic relationship between the two species. On the other hand, the genus *Hyphessobrycon* was confirmed to be a paraphyletic group, with some of its species clustering together with species from the genera *Gymnocorymbus*, *Hemigrammus*, and *Pristella*. Additionally, the phylogenetic analysis supported the monophyly of several genera, including *Moenkhausia*, *Megalamphodus*, and *Psalidodon*. This study provides new molecular evidence for the taxonomic revision and germplasm resource evaluation of the family Acestrorhamphidae. However, the lack of whole‐genome data, particularly for the genus *Moenkhausia*, remains a bottleneck, hindering in‐depth research on adaptive evolution and speciation mechanisms. Future efforts should systematically conduct whole‐genome sequencing and integrated analyses to enhance the understanding of the phylogeographic patterns and evolutionary history of this group.

## Graphical abstract

This study successfully decoded the complete mitochondrial genomes of two Moenkhausia species, with total lengths of 16,439 and 16,461 bp, respectively. This study enhances genomic resources for Acestrorhamphidae and provides a foundation for investigating evolutionary mechanisms and developing molecular markers.

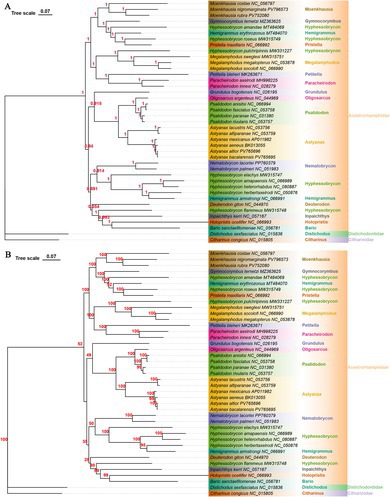

## 1 Introduction

The suborder Characoidei, a highly diverse group within the order Characiformes of the class Actinopterygii, is widely distributed in freshwater basins of Central and South America and Africa, such as the Amazon and Congo Rivers. This suborder comprises approximately 20 families (Melo et al. 2021), including Acestrorhamphidae, Alestiidae, and Serrasalmidae, and exhibits remarkable morphological and ecological diversity. Species within this group range from small ornamental fish to large food fish. Most species possess ctenoid scales, a complete lateral line, and an adipose fin, with dental morphology often specialized to adapt to various feeding habits. Their ecological behaviors are equally diverse: they inhabit still or slow‐flowing waters, exhibit carnivorous, herbivorous, or omnivorous diets, and display reproductive strategies ranging from egg‐guarding behaviors to seasonal migrations (Lavoué et al. 2017). Characoidei species hold significant economic and ornamental value, serving as important food fish resources in South America and playing a prominent role in the global aquarium trade (Domingos et al. 2014). However, some species can become invasive when introduced to non‐native regions, posing threats to local ecosystems (Trujillo‐González et al. 2018; Saba et al. 2020; Borges et al. 2022). The family Acestrorhamphidae within the suborder Characoidei, a group of freshwater fish characterized by their adipose fins and specialized dentition, has long posed significant challenges in phylogenetic research (Marinho and Langeani 2016). The family Acestrorhamphidae within the order Characiformes is primarily distributed in freshwater basins of South America, encompassing multiple genera such as *Acestrorhynchus*, *Moenkhausia*, and *Hyphessobrycon*. Fishes of this family exhibit considerable morphological diversity, and the phylogenetic relationships within certain genera have long been contentious, with topological structures failing to be consistently resolved across different studies. Clarifying the phylogenetic relationships among these genera remains a central issue in this field (Mariguela et al. 2013; Reia et al. 2019). Despite their utility for species identification and phylogeographic studies, conventional mitochondrial markers like *COI*, *Cytb*, and D‐loop lack sufficient phylogenetic informativeness to resolve the ambiguous evolutionary relationships within this group. This is particularly evident at the genus and species levels, where convergent evolution of morphological features and transcontinental relationships between South American and African taxa have not been adequately resolved (Chakrabarty et al. 2017). Molecular studies have shown that the family may have undergone rapid radiation evolution—an evolutionary model in which the early ancestors of the Characoidei family (lipocyprinoidea) rapidly differentiated into multiple lineages in a relatively short geological time scale. As a result, the support rate of topological structure based on a single mitochondrial marker is low, and it conflicts with the morphological classification scheme (Calcagnotto et al. 2005; Nakatani et al. 2011).

The genus *Moenkhausia*, within the family Acestrorhamphidae of the suborder Characoidei, is a widely distributed group of freshwater fish native to South America. They are commonly found in slow‐moving or stagnant water bodies across the Amazon Basin, the Paraguay River, and the Guiana Shield. Currently, over 70 species have been described under this genus, but significant challenges persist in its systematics: the boundaries between multiple species groups remain ambiguous, and morphological characteristics exhibit high levels of convergence or intraspecific variation, making stable classification difficult using traditional morphology alone (Reia et al. 2019). In recent years, molecular approaches (such as mitochondrial *COI* and nuclear gene markers) have been gradually introduced but have yet to fully resolve controversies surrounding species delimitation and phylogenetic reconstruction. This is particularly true for phenotypically highly similar cryptic species or species complexes, which require further integration of multi‐dimensional data for clarification (Petrolli and Benine 2015; Marinho and Langeani 2016). Additionally, some species within this genus are sensitive to water quality changes (e.g., dissolved oxygen, pH, and organic pollutants). Against the backdrop of increasing habitat fragmentation and water eutrophication, their wild populations are facing growing survival pressures. However, systematic assessments of population size, distribution ranges, and threat status remain lacking for most species, hindering the development of effective conservation strategies (Domingos et al. 2014). Thus, while the genus *Moenkhausia* exemplifies the rich diversity of the Characoidei suborder, it also highlights core challenges such as taxonomic confusion and insufficient ecological conservation research.

This study sequenced the complete mitochondrial genomes of *Moenkhausia nigromarginata* and *Moenkhausia rubra*, and conducted a comparative analysis and genetic divergence analysis by integrating mitochondrial genomes data from 37 species of the suborder Characoidei (family Acestrorhamphidae) and two species of the suborder Citharinoidei obtained from GenBank. The research included comparisons of mitochondrial genome structural characteristics, assessments of sequence variations in protein‐coding genes (PCGs) and rRNA genes, and the construction of high‐resolution phylogenetic trees using maximum likelihood (ML) and Bayesian inference (BI) methods to explore the systematic position of *Moenkhausia* species within the family Acestrorhamphidae and their phylogenetic relationships with other taxa. This study provides new molecular evidence and data support for germplasm resource identification, optimization of systematic classification frameworks, and reconstruction of evolutionary history in the family Acestrorhamphidae.

## 2 Materials and Methods

### 2.1 Sample Collection and Sequencing

Fish were collected from the Nanjing Confucius Temple Flower, Bird, Fish, and Insect Market in Jiangsu Province. The fish samples are currently stored in the molecular laboratory of the School of life sciences, Nanjing Forestry University, and the sample numbers are S0 and y42, respectively. Under sterile conditions, pectoral fins and muscle tissues were carefully excised and preserved in anhydrous ethanol (97%) for subsequent molecular analysis. Genomic DNA extraction was performed from 100 mg of tissue using a commercial DNA isolation kit from Takara Biomedical Technology (Beijing) Co. Ltd., with quality assessment conducted through dual verification methods: 1% agarose gel electrophoresis for integrity evaluation and ultra‐micro nucleic acid protein analysis for precise quantification of concentration and purity. The amplicon sequencing of samples S0 and Y42 was conducted by Nanjing Qingke Biological Company. Based on BLAST alignment analysis via the NCBI database, sample S0 was identified as *M. nigromarginata* , while sample Y42 was confirmed as *M. rubra* . This molecular identification clarified the taxonomic status of the two samples. The sequencing phase was conducted employing established high‐throughput methodologies as described by Alexander et al. (2013). The technical process encompassed library preparation using the TruSeq Nano kit system, involving ultrasonic fragmentation of DNA to generate 300–500 bp inserts and sequenced to a depth of 13 × (10G), followed by sequential enzymatic processing for end‐repair, adenine tailing, and adapter ligation. Final sequencing was executed on the Illumina NovaSeq 6000 platform with PE150 paired‐end configuration to generate high‐fidelity genomic data.

### 2.2 Mitochondrial Genome Assembly, Annotation, and Analysis

We performed quality control and trimming of the raw sequencing data using Trimmomatic. The filtering criteria included: applying a sliding window approach (window size: 9 bp, step size: 1 bp) to truncate reads when the average quality value within the window dropped to ≤ 20; removing sequences containing more than 5 N bases; and retaining high‐quality sequences that maintained sufficient length after trimming to meet the requirements for subsequent analysis (Bolger et al. 2014). Data validation and assembly were performed using Geneious software (Kearse et al. 2012). The PCGs, tRNAs, and rRNAs were predicted and annotated using MITOS2 and MitoFish (https://mitofish.aori.u‐tokyo.ac.jp/annotation/input/), resulting in a highly accurate gene set after manual curation. Additionally, mitochondrial genomes of 39 species (Table 1) from the family Acestrorhamphidae were retrieved from GenBank as reference sequences for comparative analysis. Subsequent analyses were carried out using platforms including PhyloSuite v1.2.3 (Zhang et al. 2020), DnaSP 6 (Librado and Rozas 2009), and MEGA 11 (Kumar et al. 2018). Key analyses included: codon usage bias assessment of PCGs in the two tetra species; comparative examination of mitochondrial genome structure, length, and gene arrangement across multiple species; evaluation of mutation rates, genetic distances (p‐distance), and base composition; and genome‐wide site variation analysis of 39 Acestrorhamphidae species (including the two studied species and 37 external species) using nucleotide diversity (Pi) as a variation indicator. Ka/Ks and relative synonymous codon usage (RSCU) analyses were performed using MEGA 11 (Kumar et al. 2018).

**TABLE 1:** Samples used in this study.

Suborder	Family	Species	Size (bp)	AT%	Accession no.
Characoidei	Acestrorhamphidae	*Moenkhausia nigromarginata*	16,439	58.6	PV796573
		*Moenkhausia rubra*	16,461	59.7	PV752080
		*Astyanax altior*	16,766	58.9	PV765696
		*Astyanax bacalarensis*	16,769	58.9	PV765695
		*Nematobrycon lacortei*	17,585	62.3	PP760379
		*Hyphessobrycon heterorhabdus*	17,021	58.6	NC_080887
		*Hemigrammus armstrongi*	16,789	58.4	NC_066991
		*Megalamphodus socolofi*	17,132	58.6	NC_066990
		*Hyphessobrycon amapaensis*	17,824	59.5	NC_066989
		*Holopristis ocellifer*	18,141	60.3	NC_066993
		*Psalidodon anisitsi*	16,920	57.4	NC_066994
		*Pristella maxillaris*	16,896	57.6	NC_066992
		*Inpaichthys kerri*	17,032	60.4	NC_057167
		*Moenkhausia costae*	15,811	54.7	NC_056797
		*Bario sanctaefilomenae*	18,437	60.0	NC_056781
		*Megalamphodus megalopterus*	16,773	59.5	NC_053878
		*Astyanax altiparanae*	16,730	58.3	NC_053759
		*Psalidodon fasciatus*	16,626	57.4	NC_053758
		*Psalidodon rivularis*	16,779	57.0	NC_053757
		*Astyanax lacustris*	16,763	58.2	NC_053756
		*Nematobrycon palmeri*	17,340	61.2	NC_051983
		*Hyphessobrycon herbertaxelrodi*	17,417	59.7	NC_050876
		*Deuterodon giton*	16,643	59.2	NC_044970
		*Oligosarcus argenteus*	16,711	57.6	NC_044969
		*Psalidodon paranae*	16,707	57.1	NC_031380
		*Paracheirodon innesi*	16,759	58.4	NC_028279
		*Grundulus bogotensis*	17,123	60.1	NC_026195
		*Hyphessobrycon pulchripinnis*	17,618	57.5	MW331227
		*Hyphessobrycon roseus*	17,046	56.9	MW315749
		*Hyphessobrycon flammeus*	16,008	59.7	MW315748
		*Hyphessobrycon elachys*	17,224	59.3	MW315747
		*Megalamphodus sweglesi*	16,080	56.0	MW315751
		*Gymnocorymbus ternetzi*	17,999	58.3	MZ363625
		*Hemigrammus erythrozonus*	16,710	57.5	MT484070
		*Hyphessobrycon amandae*	16,701	57.2	MT484069
		*Astyanax aeneus*	16,769	58.8	BK013055
		*Petitella bleheri*	17,021	58.4	MK263671
		*Paracheirodon axelrodi*	17,100	59.0	MH998225
		*Astyanax mexicanus*	16,682	58.8	AP011982
Citharinoidei	Citharinidae	*Citharinus congicus*	16,453	53.7	NC_015805
	Distichodontidae	*Distichodus sexfasciatus*	16,555	57.0	NC_015836

### 2.3 Phylogenetic Analysis

To elucidate the phylogenetic relationships within the family Acestrorhamphidae, this study analyzed complete mitochondrial genome sequences of 37 species from this family (Table 1) obtained from the GenBank database, with *Citharinus congicus* (NC_015805) and *Distichodus sexfasciatus* (NC_015836) selected as outgroups (Nakatani et al. 2011). Phylogenetic trees were constructed based on the concatenated sequences of 13 mitochondrial PCGs. Initially, sequences were aligned using MAFFT v7.313 (Katoh et al. 2002), followed by codon site correction with MACSE v2.03 (Ranwez et al. 2018). To integrate evolutionary signals and resolve topological instability caused by limited informative sites in single genes, all 13 PCGs were concatenated into a single supermatrix to obtain a more robust phylogenetic tree. To account for evolutionary heterogeneity among different genes, we employed a partitioned analysis strategy rather than reconstructing separate trees. The optimal partition models were selected via ModelFinder (Kalyaanamoorthy et al. 2017) based on the Bayesian Information Criterion (BIC). For the BI analysis in MrBayes v3.2.6 (Ronquist et al. 2012), the majority of genes followed the GTR + F + I + G4 model, while *ATP8* and *ND2* were specifically assigned the HKY + F + I + G4 model within the partitioned framework. The BI analysis was conducted with Markov chains running for 10 million generations and a 25% burn‐in proportion. For the ML analysis performed with IQ‐TREE v1.6.8 (Nguyen et al. 2015), different gene partitions were assigned various models, including K3Pu + F + R4, GTR + F + I + I + R4, and TIM2 + F + R5, using 10,000 UFBoot bootstrap replicates. Finally, the phylogenetic trees were visualized using iTOL v6 (Letunic and Bork 2007).

## 3 Results

### 3.1 Characteristics of the Mitochondrial Genome

**FIGURE 1:**
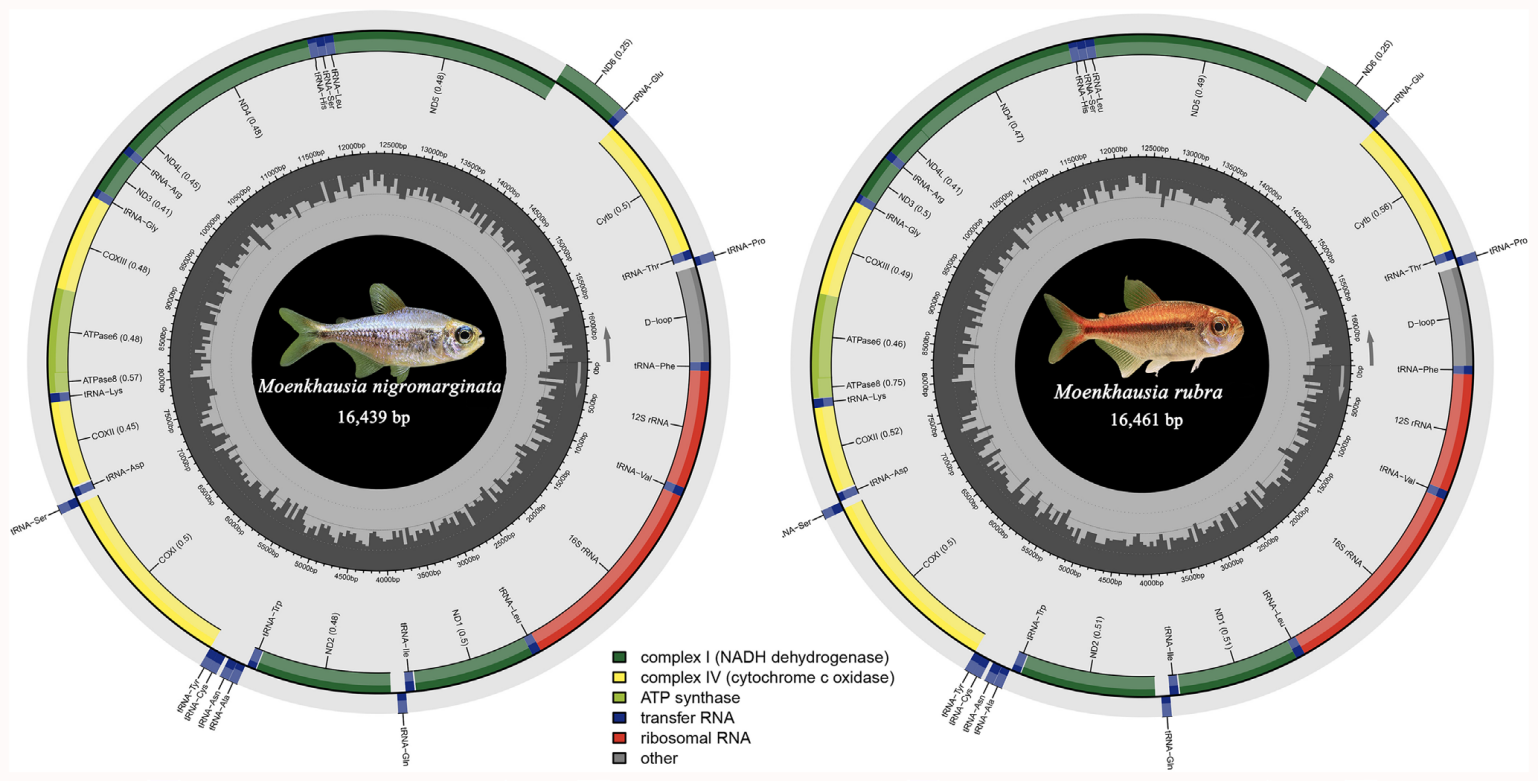
Circular maps of the mitochondrial genomes of two fish species, illustrating the distribution and organization of mitochondrial genes. The left panel represents *M. nigromarginata* with a genome size of 16,439 bp, while the right panel represents *M. rubra* with a genome size of 16,461 bp. The circular structure annotates genes by functional categories: Yellow (cytochrome c oxidase subunits, Complex IV), green (NADH dehydrogenase subunits, Complex I), gray (ATP synthase subunits), blue (cytochrome b), red (ribosomal RNAs), purple (transfer RNAs), and others.

The complete mitochondrial genome sequences of *M. nigromarginata* and *M. rubra* obtained in this study have been annotated and submitted to GenBank (accession numbers: PV796573 and PV752080, respectively). The mitochondrial genomes exhibit a typical circular structure (Figure 1), with total lengths of 16,439 and 16,461 bp, respectively. The combined length of the 13 PCGs is 11,421 and 11,448 bp, accounting for 69.48% and 69.55% of the total mitochondrial genome length, respectively. The 22 tRNAs have a combined length of 1553 bp and 1550 bp, representing 9.45% and 9.42% of the total genome length, respectively. Except for eight tRNAs (*tRNA‐Asn*, *tRNA‐Cys*, *tRNA‐Gln*, *tRNA‐Tyr*, *tRNA‐Ser*, *tRNA‐Glu*, *tRNA‐Pro*, and *tRNA‐Ala*), the remaining 14 tRNAs are located on the heavy strand. The *12S rRNA*, located between *tRNA‐Phe* and *tRNA‐Val*, is 952 bp in length, while the *16S rRNA*, situated between *tRNA‐Val* and *tRNA‐Leu*, is 1675 bp in length (Table 2).

**TABLE 2:** Annotation of the complete mitochondrial genome of *Moenkhausia nigromarginata* and *Moenkhausia rubra*.

Gene	Position		Size (bp)	Intergenic nucleotides	Codon		Strand
	From	To			Start	Stop	
*tRNA‐Phe*	1/1	70/68	70/68				H
*12S rRNA*	71/69	1022/1021	952/953				H
*tRNA‐Val*	1023/1022	1094/1093	72/72				H
*16S rRNA*	1095/1094	2769/2767	1675/1674				H
*tRNA‐Leu*	2770/2768	2844/2842	75/75				H
*ND1*	2845/2843	3813/3811	969/969		ATG	TAA	H
*tRNA‐Ile*	3825/3823	3896/3894	72/72	11/11			H
*tRNA‐Gln*	3895/3893	3965/3963	71/71	−2/−2			L
*tRNA‐Met*	3975/3972	4040/4036	66/65	9/8			H
*ND2*	4042/4011	5100/5096	1059/1086	1/−26	ATG	TAA	H
*tRNA‐Trp*	5109/5105	5179/5177	71/73	8/8			H
*tRNA‐Ala*	5194/5190	5262/5258	69/69	14/12			L
*tRNA‐Asn*	5264/5260	5336/5332	73/73	1/1			L
*tRNA‐Cys*	5367/5364	5433/5430	67/67	30/31			L
*tRNA‐Tyr*	5433/5430	5503/5500	71/71	‐1/−1			L
*COXI*	5505/5502	7061/7058	1557/1557	1/1	GTG	AGG	H
*tRNA‐Ser1*	7049/7046	7120/7117	72/72	−13/−13			L
*tRNA‐Asp*	7124/7121	7194/7190	71/70	3/3			H
*COXII*	7209/7205	7896/7892	688/688	14/14	ATG	T	H
*tRNA‐Lys*	7897/7893	7969/7965	73/73				H
*ATPase8*	7973/7969	8140/8136	168/168	3/3	ATG	TAA	H
*ATPase6*	8131/8127	8812/8808	682/682	−10/−10	GTG	T	H
*COXIII*	8813/8809	9596/9592	784/784		ATG	T	H
*tRNA‐Gly*	9597/9593	9666/9662	70/70				H
*ND3*	9667/9663	10,015/10,011	349/349		ATG	T	H
*tRNA‐Arg*	10,016/10,012	10,084/10,080	69/69				H
*ND4L*	10,085/10,081	10,381/10,377	297/297		ATG	TAA	H
*ND4*	10,375/10,371	11,755/11,751	1381/1381	−7/−7	ATG	T	H
*tRNA‐His*	11,756/11,752	11,824/11,820	69/69				H
*tRNA‐Ser2*	11,825/11,821	11,892/11,887	68/67				H
*tRNA‐Leu2*	11,894/11,889	11,966/11,961	73/73	1/1			H
*ND5*	11,967/11,962	13,805/13,800	1839/1839		ATG	TAA	H
*ND6*	13,802/13,797	14,317/14,312	516/516	−4/−4	ATG	TAA	L
*tRNA‐Glu*	14,318/14,313	14,385/14,380	68/68				L
*Cytb*	14,389/14,384	15,525/15,520	1137/1137	3/3	ATG	AGG/AGA	H
*tRNA‐Thr*	15,530/15,525	15,602/15,597	73/73	4/4			H
*tRNA‐Pro*	15,601/15,596	15,670/15,665	70/70	−2/−2			L
D‐loop	15,671/15,666	16,439/16,461	769/796				

**FIGURE 2:**
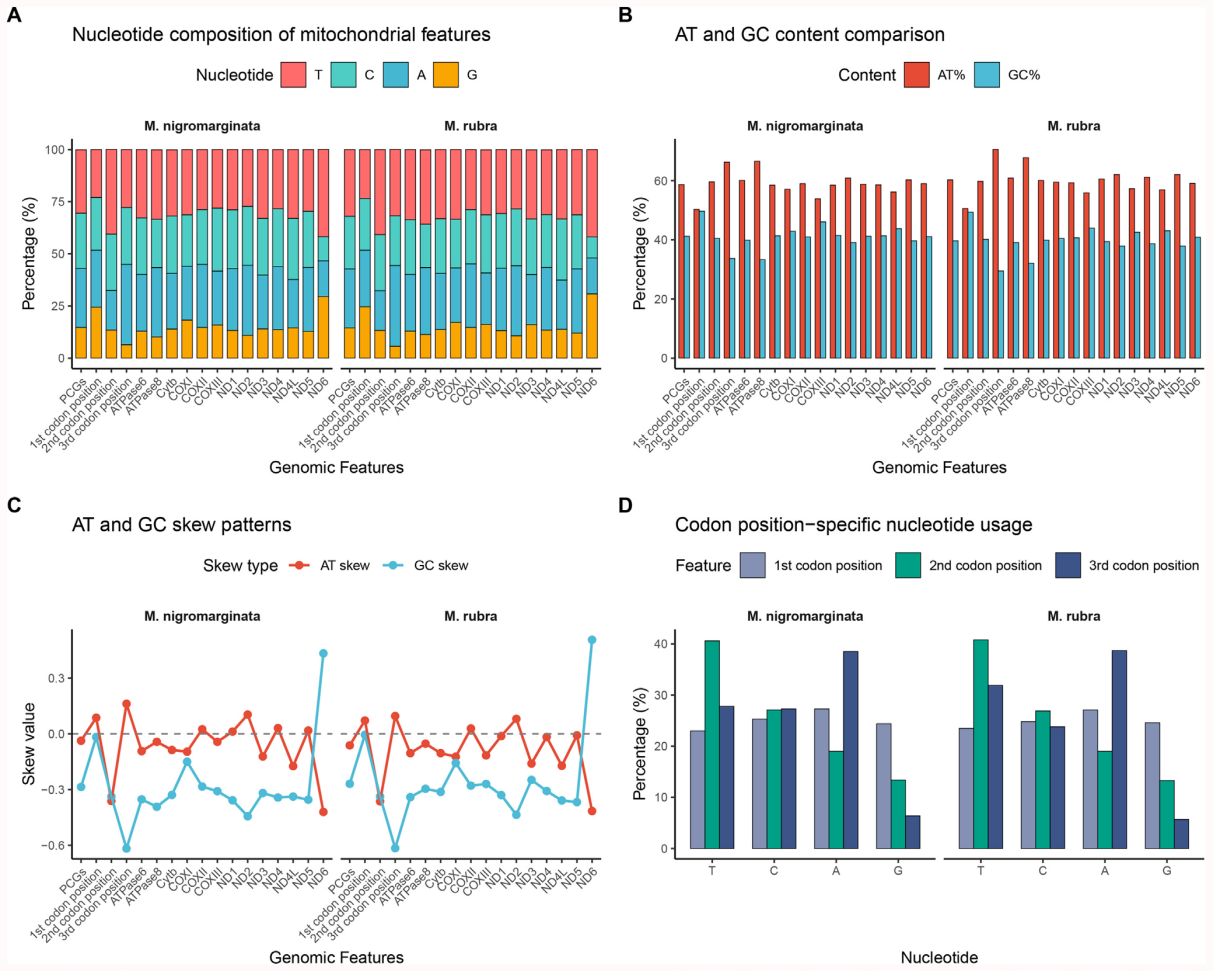
(A) Nucleotide composition of mitochondrial genomic features: This panel displays the nucleotide composition (T, C, A, G) of various genomic features, including genes and non‐coding regions, in the mitochondrial genomes of *M. nigromarginata* and *M. rubra* . The horizontal axis (“Genomic Features”) represents specific elements, with the nucleotide percentages illustrating the compositional differences between the two species. (B) AT and GC content comparison: This section illustrates the AT% and GC% content of various genomic features in the mitochondrial genomes of both species, highlighting the AT/GC biases and differences in nucleic acid composition. (C) AT and GC skew patterns: This part presents the AT‐skew and GC‐skew values for various genomic features, reflecting base bias trends that are influenced by molecular processes such as replication and repair. (D) Codon position‐specific nucleotide usage: This panel provides statistics on the frequency of nucleotide usage (T, C, A, G; where “f” denotes U, accounting for the special nature of the mitochondrial genetic code) at the first, second, and third positions of codons, revealing nucleotide preferences specific to each codon position.

The base composition of *M. nigromarginata* and *M. rubra* was determined as (T‐28%, C‐26.1%, A‐30.6%, G‐15.2%) and (T‐29.2%, C‐25.2%, A‐30.5%, G‐15%), respectively. The proportional distribution of the four nucleotides (T, C, A, G) was relatively similar between the two species but exhibited subtle differences. *M. nigromarginata* showed slightly higher T content, while *M. rubra* had relatively higher A content, indicating subtle variations in the base composition of their mitochondrial genomes (Figure 2A). The AT content was significantly higher than the GC content in both species: *M. nigromarginata* (58.6% > 41.3%) and *M. rubra* (59.7% > 40.2%), demonstrating a clear AT bias. The AT content in both species was significantly higher than the GC content, consistent with the general characteristic of high AT content in animal mitochondrial genomes. *M. rubra* exhibited slightly higher AT content and slightly lower GC content compared to *M. nigromarginata* , suggesting its mitochondrial genome may have a slightly more pronounced AT bias (Figure 2B). Additionally, the AT skew and GC skew values were close to zero in both species but showed minor fluctuations. *M. nigromarginata* had a slightly negative AT skew, indicating a slight predominance of T over A on the strand, while the GC skew was slightly positive, indicating a slight predominance of G over C. M. rubra exhibited a similar skew pattern but with different magnitudes, reflecting strand‐specific base usage differences (Figure 2C). Across the three codon positions, both species showed consistent nucleotide usage trends: the third position exhibited the highest variability with higher A/T usage, consistent with the degeneracy of the mitochondrial genetic code, while the first and second positions were relatively conserved. *M. rubra* had slightly higher A/T usage at the third codon position compared to *M. nigromarginata* , which may be related to translational efficiency or natural selection pressures (Figure 2D).

### 3.2 Sequence Analysis

**FIGURE 3:**
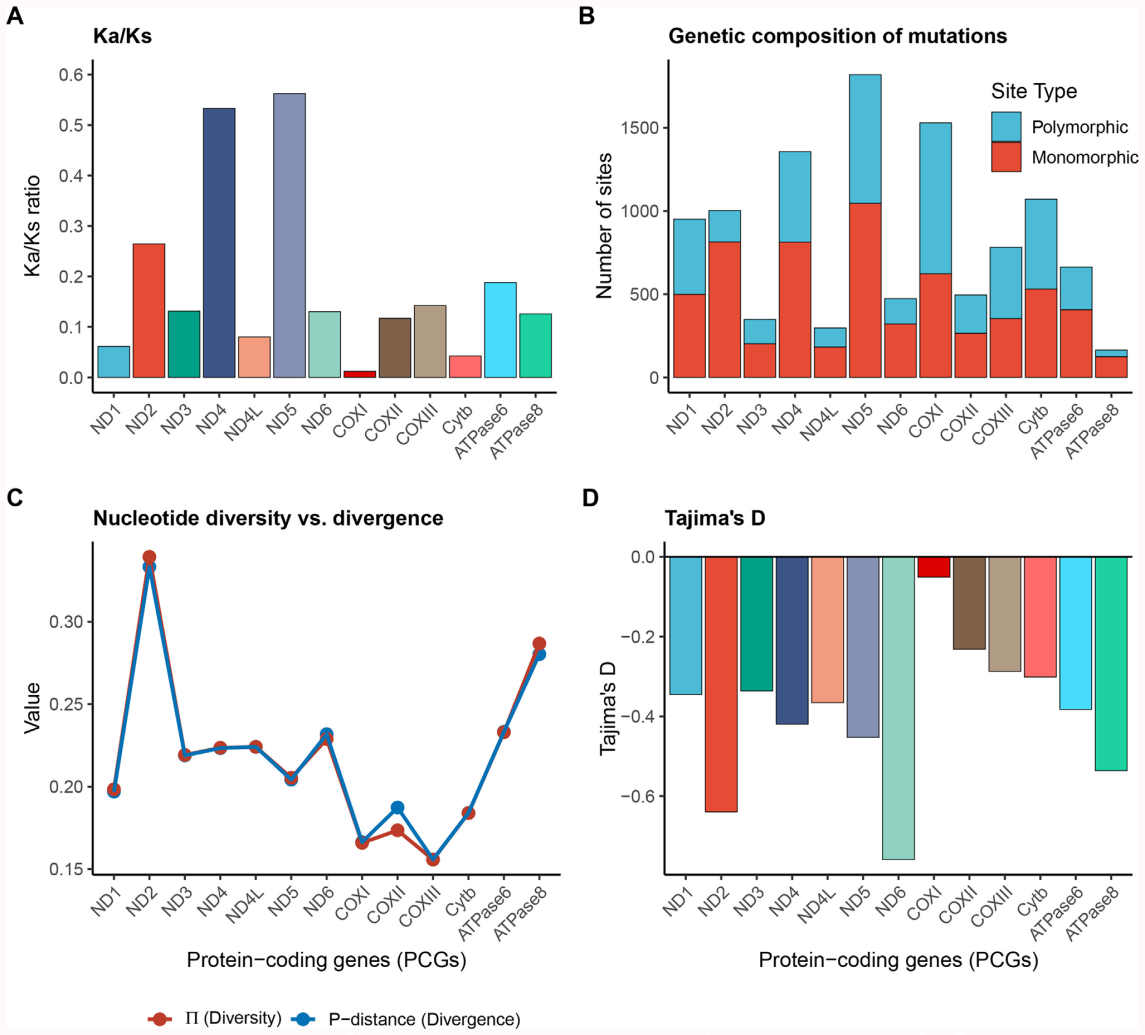
Evolutionary characteristics of mitochondrial PCGs in the family Acestrorhamphidae. (A) Selection pressure (Ka/Ks) analysis: Bar plots display the Ka/Ks ratio for each gene, reflecting the effects of purifying selection or positive selection. (B) Genetic composition of mutations: Stacked bar charts compare the number of polymorphic and monomorphic sites per gene, illustrating the potential for genetic variation. (C) Association between nucleotide diversity (Pi, red) and divergence (P‐distance, blue): Line graphs depict gene‐level differences in intraspecific diversity and interspecific divergence. (D) Tajima's *D* statistic: Bar plots show Tajima's *D* values for each gene, distinguishing signals of selection or demographic dynamics related to population genetic structure.

The Ka/Ks ratios varied significantly among different PCGs, with *COI* exhibiting the lowest value (0.012) and *ND5* the highest (0.56). All 13 PCG genes had Ka/Ks < 1, indicating overall purifying selection constraints. However, genes such as *ND3* and *ND4L* showed relatively higher Ka/Ks values, suggesting weaker purifying selection or potential signals of adaptive evolution, reflecting the influence of gene functional differences on selective pressures (Figure 3A). The analysis revealed significant heterogeneity in evolutionary rates among the 13 PCGs (Figure 3B). Specifically, *ND5* and *COX1* possessed the highest absolute numbers of polymorphic sites (1046 and 623, respectively), while the proportion of polymorphic sites was highest in *ATP8* (124 sites, 75.6%) and *ATP6* (407 sites, 61.4%), indicating rapid variation accumulation in these genes. In contrast, *COXIII* and *ND4L* exhibited relatively higher proportions of invariable sites (54.9% and 38.7% respectively), reflecting stronger functional constraints.

Furthermore, nucleotide diversity (Pi) and P‐distance fluctuated across genes, ranging from 0.15588 (*COXIII*) to 0.28035 (*ATP8*). The consistently low Pi values in the COX gene family suggest they are under intense purifying selection due to their critical role in mitochondrial metabolism. Accordingly, nucleotide diversity (Pi) and P‐distance fluctuated across genes, ranging from 0.15588 (*COXIII*) to 0.28035 (*ATP8*) (Figure 3C). The consistently low Pi values observed in the COX gene family are closely related to their core roles in mitochondrial energy metabolism, suggesting that they have been subject to intense purifying selection. Conversely, the high levels of diversity exhibited by the *ATP8* and ND family genes indicate their potential utility as molecular markers for interspecific differentiation.

Tajima's *D* values for all 13 PCGs were negative, ranging from −0.05098 (*COX1*) to −0.53629 (*ATP8*) (Figure 3D). Although these negative values were not statistically significant (*p* > 0.10), the consistent negative trend suggests an excess of low‐frequency polymorphisms within the population. The most plausible biological scenario for this pattern is that intense purifying selection has maintained the functional integrity of the genomes in *M. nigromarginata* and *M. rubra* , or that these two species underwent a recent population expansion following an evolutionary bottleneck.

**FIGURE 4:**
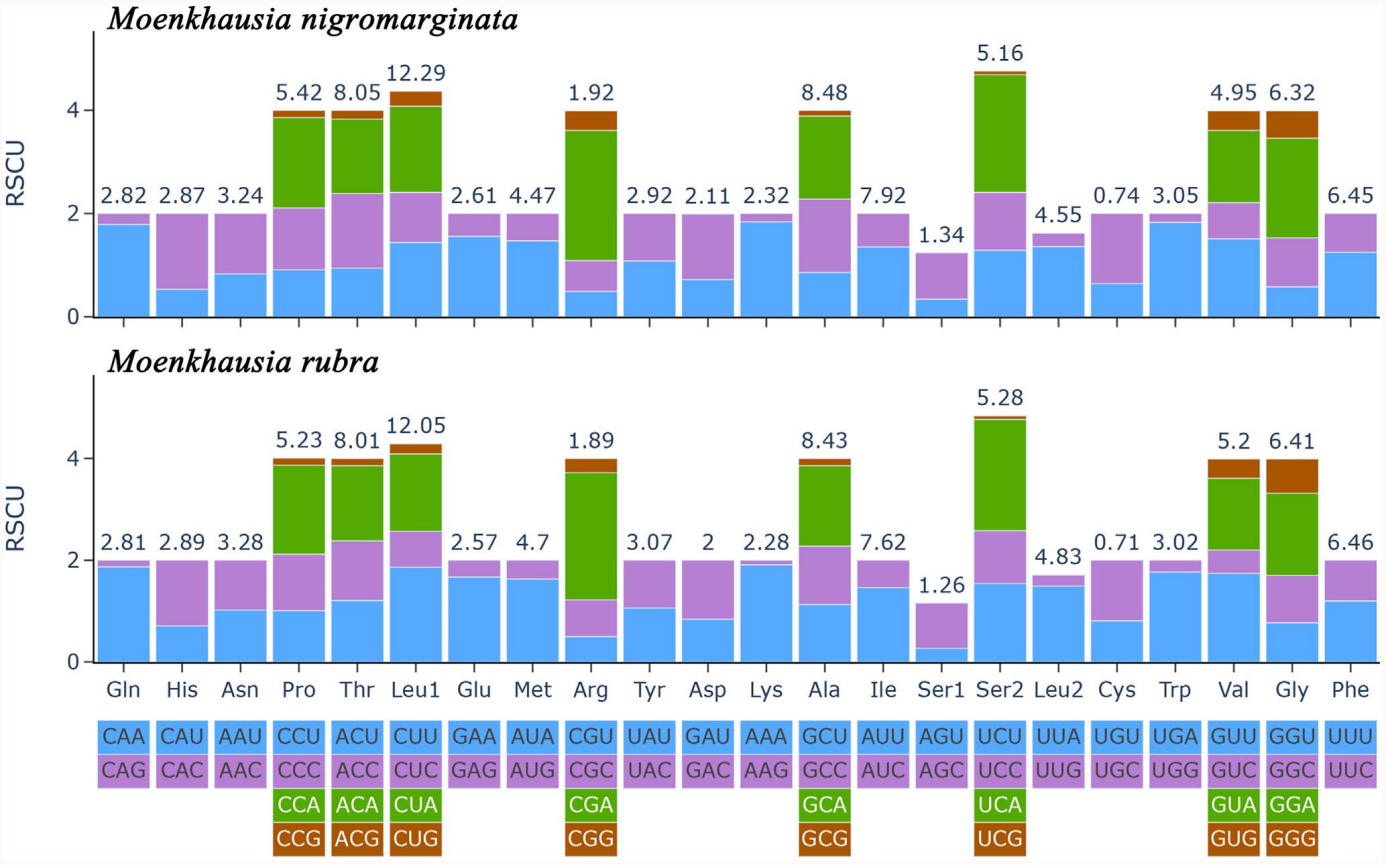
Relative Synonymous Codon Usage (RSCU) in *M. nigromarginata* and *M. rubra* . The upper and lower sections correspond to the two fish species, respectively. The horizontal axis represents codons and their encoded amino acids, while the vertical axis (RSCU) reflects codon usage bias. Bars of different colors denote different codons for the same amino acid, with numerical labels indicating frequency differences. This analysis elucidates the codon preference patterns in the mitochondrial genes of the two species.

The Figure 4 displays RSCU in the mitochondrial genomes of two fish species, *M. nigromarginata* and *M. rubra* . The codon usage patterns of these two species exhibit both highly conserved commonalities and species‐specific differences. Codons such as CUU (encoding leucine, Leu) and ACU (encoding threonine, Thr) show high RSCU values in both species, indicating preferred usage, while codons like CGG (encoding arginine, Arg) generally exhibit low RSCU values. Simultaneously, subtle interspecific differences in RSCU values are observed for certain codons, such as UCU (encoding serine, Ser1) and AAA (encoding lysine, Lys). Additionally, both species exhibit a distinct distribution of high‐RSCU codons and low‐RSCU codons. The former may align with the demand for efficient mitochondrial translation, while the latter may be constrained by low tRNA abundance or poor translational efficiency. This demonstrates the optimization strategies of mitochondrial genes in codon selection to balance energy consumption and translation rates, ensuring functional stability and metabolic efficiency.

### 3.3 Phylogenetic Analysis

**FIGURE 5:**
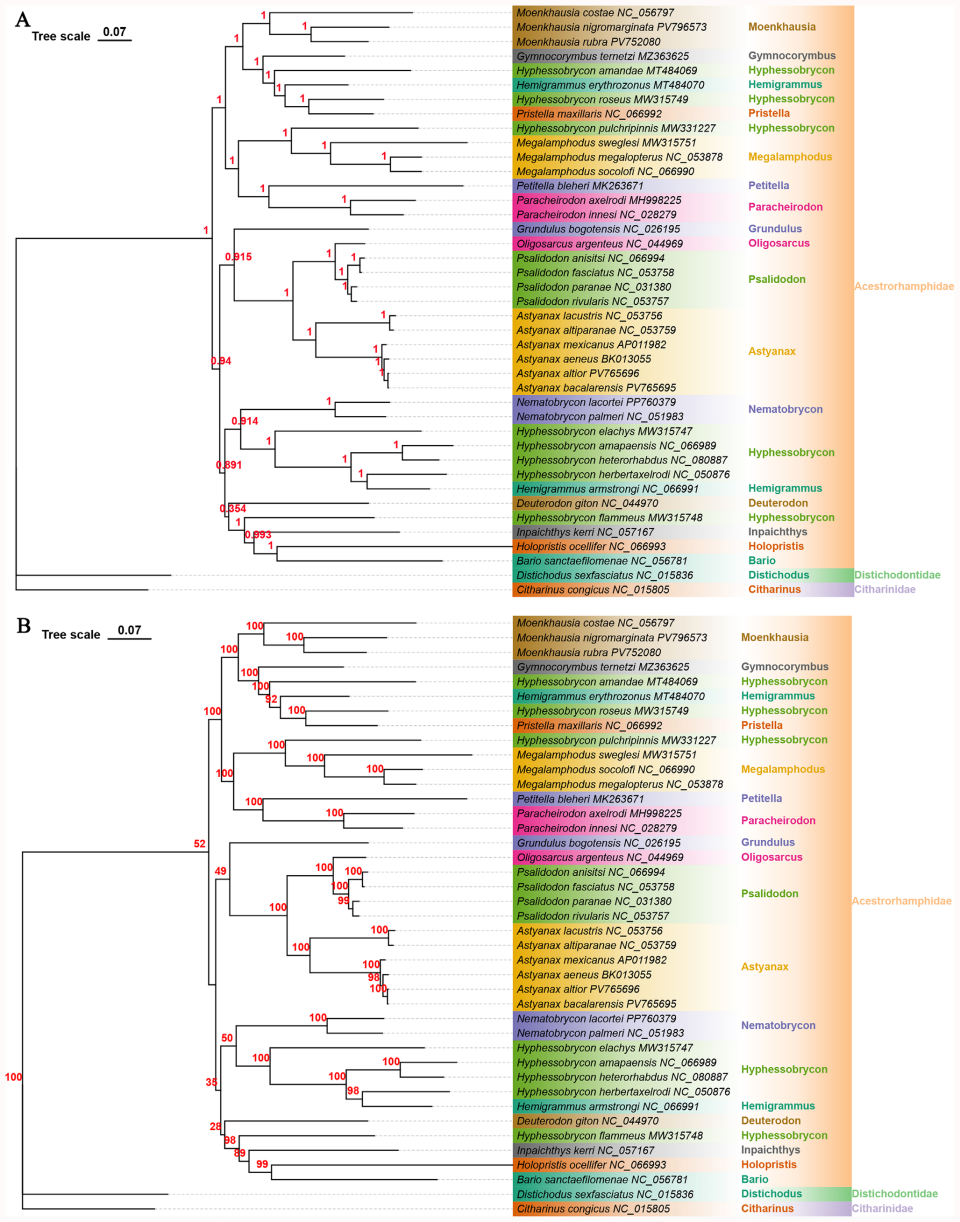
Phylogenetic tree illustrating the evolutionary relationships within the family Acestrorhamphidae and related taxa, reconstructed based on mitochondrial genome data. (A) Bayesian inference (BI) tree; (B) Maximum likelihood (ML) tree. The trees annotate taxonomic information at the family level and genus level, with distinct colors corresponding to different taxonomic groups. The branching structures and support values elucidate the phylogenetic affinities and evolutionary divergence patterns.

Using mitochondrial genomes from 41 species within the Characoidei suborder, phylogenetic trees were constructed based on concatenated sequences of 13 PCGs, including a BI tree (Figure 5A) and a ML phylogenetic tree (Figure 5B). The topological structures of both trees were highly consistent, providing robust support for the monophyly of the genera *Moenkhausia*, *Megalamphodus*, *Psalidodon, Astyanax*, *Paracheirodon*, and *Nematobrycon* (Bayesian posterior probability [PP] = 1.00; ML bootstrap support [BS] = 100). Within the genus *Moenkhausia*, *M. nigromarginata* and *M. rubra* formed a well‐supported monophyletic clade with maximum support values (PP = 1.00, BS = 100). Notably, *M. nigromarginata* and *M. rubra* exhibited a closer phylogenetic relationship to each other than to *Moenkhausia costae* , a clustering pattern that was also strongly recovered by both inference methods (PP = 1.00, BS = 100).

## 4 Discussion

Fish of the family Acestrorhamphidae serve as significant components of South American freshwater ecosystems. As members of the subfamily Stichonodontinae, species in the genus *Moenkhausia* are typically distributed in the middle and upper layers of water bodies, where their presence is closely associated with hydrological dynamics and aquatic vegetation coverage. Fluctuations in the population size of these Acestrorhamphids can effectively reflect the environmental health and biodiversity status of Neotropical aquatic systems (Lima et al. 2025). Recent phylogenomic studies using high‐throughput sequencing have significantly refined the classification of this group (Melo et al. 2024), providing a robust framework for understanding the evolutionary history of Moenkhausia and its relatives.

The complete mitochondrial genomes of *M. nigromarginata* and *M. rubra* were successfully assembled in this study. Both genomes exhibited a typical circular structure with similar total lengths (Alvarenga et al. 2024). Analysis of gene composition revealed that the 13 PCGs constituted the major portion of the genomes, accounting for approximately 69.5% of the total length. Base composition analysis demonstrated a significant AT bias in both species, reflecting a stronger base composition bias in the former. Codon usage patterns were highly conserved, with codons such as CUU and ACU being frequently used. However, interspecific differences were observed in the usage frequency of certain codons. This variation is consistent with the action of natural selection fine‐tuning translational efficiency, a phenomenon widely documented in genomic studies (Satoh et al. 2016). Positive Tajima's *D* values may indicate balancing selection or influences from pre‐expansion population history, leading to unique patterns of variation accumulation. Negative values suggest purifying selection or population expansion, reflecting differences in the evolutionary responses of genes to population dynamics and selective pressures (Moura et al. 2014). In summary, while the basic structure of the mitochondrial genomes remains conserved between the two species, interspecific differences in base composition and codon usage provide reliable molecular evidence for their valid taxonomic separation.

Our phylogenetic analysis robustly demonstrates that *M. nigromarginata* and *M. rubra* form a well‐supported monophyletic clade, confirming their close phylogenetic relationship within the genus *Moenkhausia* (Rodrigues‐Oliveira et al. 2023). This finding is consistent with their shared morphological characteristics and provides a solid molecular framework for their taxonomic affiliation (Mariguela et al. 2013). The monophyly of the genus *Moenkhausia* received strong support in our analysis, a finding that aligns with the broader phylogenetic patterns observed in some molecular studies focusing on characids (Reia et al. 2019). Congruent with previous comprehensive phylogenies, our results further substantiate that the genus *Hyphessobrycon* is a paraphyletic group (Verboom et al. 2020; van den Ende et al. 2023). Specifically, we observed that species historically assigned to Hyphessobrycon were interspersed within clades predominantly composed of Moenkhausia and Hemigrammus species. This pattern of lineage intermingling has been consistently reported in larger‐scale phylogenetic studies of the Characidae family. For instance, Mirande (2010) and more recent works utilizing high‐throughput data (Ota et al. 2020) have highlighted the complex evolutionary network and the polyphyletic nature of Hyphessobrycon. The primary driver behind this systematic discrepancy is widely considered to be convergent evolution, where traditional morphological traits used for classification have evolved independently in distinct lineages adapting to similar ecological pressures or selective forces (Hu et al. 2023). While most relationships were robustly resolved, some nodes—such as the placement of *Grundulus* relative to the *Psalidodon–Astyanax* clade and the affinity between the *Hyphessobsohycon–Inpaichthys–Holopristis–Bario* group and *Deuterodon*—exhibited methodological inconsistencies or low support, potentially reflecting incomplete lineage sorting or historical hybridization (Schaefer et al. 2016; Feng et al. 2022; Liu and Zhao 2023). These results underscore the need for integrated mitogenomic and multi‐locus nuclear data to refine generic boundaries and resolve deep relationships within the family.

## 5 Conclusion

This study successfully decoded and annotated the complete mitochondrial genomes of *Moenkhausia nigromarginata* and *Moenkhausia rubra*. The primary objectives were to characterize their mitogenomic structures and to employ these complete sequences for phylogenetic reconstruction within the family Acestrorhamphidae. This work enhances the genomic resources for Acestrorhamphidae and provides a foundational dataset for subsequent evolutionary studies and the development of molecular markers.

## Author Contributions

**Cheng‐He Sun:** conceptualization (lead), data curation (lead), formal analysis (equal), funding acquisition (equal), methodology (lead), writing – original draft (equal), writing – review and editing (equal). **Xiao‐Die Chen:** formal analysis (equal), writing – original draft (equal), writing – review and editing (equal). **Yi‐Jing Zhan:** data curation (equal), formal analysis (equal), writing – review and editing (equal). **Yang Xu:** formal analysis (equal), writing – review and editing (equal). **Chang‐Hu Lu:** funding acquisition (equal), writing – review and editing (equal).

## Funding

This study was supported by the Priority Academic Program Development of Jiangsu Higher Education Institutions (PAPD).

## Ethics Statement

All specimens in this study were collected in full compliance with Chinese regulations and guidelines. The collection and sampling procedures were rigorously reviewed and approved by the Animal Ethics Committee of Nanjing Forestry University, ensuring strict adherence to ethical standards for animal welfare and care throughout the experimental process.

## Consent

The authors have nothing to report.

## Conflicts of Interest

The authors declare no conflicts of interest.

## Data Availability

The data presented in this study were deposited in the NCBI repository (https://www.ncbi.nlm.nih.gov, accession numbers for *Moenkhausia nigromarginata* and *Moenkhausia rubra* are PV796573 and PV752080, respectively).
